# Non-Contrast MR T2-Weighted Imaging Is as Accurate as Contrast-Enhanced T1-Weighted Imaging in the Detection of Meningioma Growth

**DOI:** 10.3390/cancers17233800

**Published:** 2025-11-27

**Authors:** Bianca M. Dijkstra, Guillaume A. Padmos, Martijn P. G. Broen, Daniëlle B. P. Eekers, Monique H. M. E. Anten, Alida A. Postma

**Affiliations:** 1 Department of Neurosurgery, Maastricht University Medical Center, 6229 HX Maastricht, The Netherlands; 2 Department of Radiology and Nuclear Medicine, Maastricht University Medical Center, 6229 HX Maastricht, The Netherlands; 3 Department of Neurology, Maastricht University Medical Center, 6229 HX Maastricht, The Netherlands; 4 Department of Radiation Oncology (Maastro), GROW School for Oncology and Reproduction, Maastricht University Medical Center +, 6229 HX Maastricht, The Netherlands; 5 Mental Health and Sciences Research Institute (Mhens), Maastricht University, 6211 LK Maastricht, The Netherlands

**Keywords:** meningioma, magnetic resonance imaging, follow-up, gadolinium

## Abstract

**Background/Objectives**: Asymptomatic meningiomas require frequent follow-up using MR imaging, with the standard of care being contrast-enhanced T1-weighted imaging (CE-T1WI) with Gadolinium-Based Contrast Agents (GBCAs). Limiting GBCA exposure reduces the environmental impact and limits possible gadolinium deposition in the brain. Therefore, the research objective was investigating the diagnostic accuracy of T2WI for evaluating significant meningioma growth (≥10% per year), using the CE-T1WI as reference standard. **Methods**: A total of 99 asymptomatic patients with the radiological diagnosis of meningioma and a minimum follow-up period of 11 months were retrospectively identified. Patients were scanned with various scanners in multiple hospitals. The maximum tumor diameter was measured in the transverse plane. Tumor growth was calculated in changes in millimeters and converted to percentages in the longest tumor diameter in the transverse plane. A paired-sample *t*-test was used to compare tumor growth on T2WI to CE-T1WI. The diagnostic accuracy of T2WI was determined by calculation of sensitivity, specificity, and positive and negative predictive values. **Results**: Mean follow-up time was 1.9 years. Significant tumor growth was found in 16 patients using T2WI, compared to 10 patients using CE-T1WI, which was not statistically significant. T2WI had a sensitivity of 80%, specificity of 90%, a positive predictive value of 47%, and negative predictive value of 98% for prediction of significant meningioma growth. **Conclusions**: T2WI was not inferior to CE-T1WI in the detection of significant tumor growth in asymptomatic meningioma and therefore can be used in follow-up to reduce gadolinium exposure.

## Simple Summary

Tumors that grow from membranes surrounding brain and spinal cord, called meningiomas, require frequent follow-up to monitor any significant changes. This study evaluates an imaging technique, called T2-weighted magnetic resonance imaging (MRI), that highlights tissues such as the brain without using dye. The ability of this imaging technique to detect meningioma growth was compared to that of a similar imaging technique, called T1-weighted MRI, which does require dye—specifically gadolinium-based contrast. Gadolinium from human sources is increasingly detected in aquatic systems worldwide as well as in body tissues; reducing its use is beneficial for the environment and may also be safer for patients. The 99 patients included in this study were followed for 1.9 years on average. Tumor growth was measured using both imaging techniques, and both correctly detected clinically significant growth. These results suggest that the dye-free imaging technique is as accurate as the technique requiring dye.

## 1. Introduction

Meningiomas are common primary extra-axial tumors of the central nervous system (CNS). The majority are asymptomatic and discovered as incidental findings [1,2,3,4]. The diagnosis of meningioma is often made based on features of radiological imaging. An extra-axial lesion with a dural tail, cerebrospinal fluid cleft, and (moderate) hyper-intense signal intensity on MR T2-weighted images (T2WI), with enhancement after gadolinium, make a likely diagnosis [5]. Active treatment through surgical resection and/or radiotherapy is indicated for symptomatic meningiomas or clinically significant tumor growth (≥10% growth per year), with frequent radiological follow-up. In asymptomatic patients, a “wait-and-scan” approach can be adapted, in which patients undergo radiological surveillance with watchful waiting [6].

Radiological follow-up is performed using standard MRI sequences such as T1-weighted images (T1WI), contrast-enhanced T1WI (CE-T1WI), T2WI, FLAIR, and diffusion weighted images. The most commonly used method for monitoring tumor growth is CE-T1WI, with the administration of gadolinium-based contrast agents (GBCAs) [6,7,8]. GBCAs are produced by chelating the rare metal gadolinium (Gd^3+^) to a chelating ligand. As most meningiomas are indolent and require years of follow-up with frequent radiological imaging, it is important to prevent exposure to GBCAs in order to reduce the environmental impact of applying Gd^3+^ and to reduce the potential risks of Gd^3+^-deposition in patients. In clinical practice, conventional sequences such as T2WI, FLAIR, and T1WI are used in conjunction with CE-T1WI. Some reports show that T2WI provides comparable results to CE-T1WI for assessing tumor growth [9,10,11]. Our current study aims to investigate whether T2WI is a non-inferior method for the detection of meningioma growth compared to CE-T1WI in untreated meningioma patients.

## 2. Materials and Methods

### 2.1. Patient Selection

This retrospective study was performed on data of patients diagnosed with an intracranial meningioma between January 2014 and January 2018. All patient records were screened in October 2025 to obtain the latest clinical evaluation and pathological analysis if available. The study was approved by the internal review board (#2018-0820). The need to obtain informed consent from each participant was waived. Research was conducted in accordance with the Declaration of Helsinki. The inclusion criteria were a minimum age of 18 years, diagnosis of meningioma, and presence of at least two cerebral T2WI and CE-T1WI MR scans within a minimum follow-up period of 11 months. The last criterion was based on the yearly interval for newly diagnosed meningioma patients to evaluate tumor growth. Patients were excluded if MR scans were incomplete regarding sequences, if the meningioma showed extracranial involvement, or if patients underwent surgical resection or radiotherapy during the follow-up time. Of note, the majority of patients received their initial diagnoses and further follow-up in the referring (secondary) center.

### 2.2. Imaging Protocol

Standard-of-care MR images were obtained with a 1.5 or 3T scanner, and basic imaging protocol at baseline and follow-up scanning consisted of conventional series, including T1WI, CE-T1WI, T2WI, and FLAIR. Only CE-T1WI and T2WI were used for this study. MRI scans from various hospitals with a variety of scanners were obtained and evaluated at our tertiary hospital. A full standard single dose of gadolinium-based contrast agent was administered intravenously prior to CE-T1WI imaging.

### 2.3. Data Interpretation

**Figure 1:**
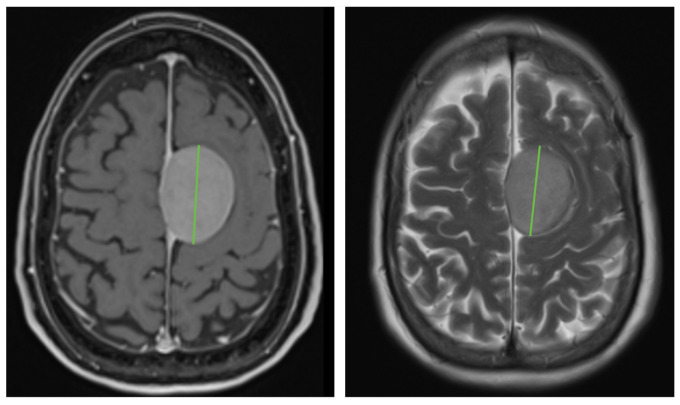
Representative example of the method of measuring meningioma size in the transverse plane of a parasagittal meningioma with a maximum diameter of 45 mm (green line) on CE-T1WI (**left**) and T2WI (**right**).

The MR images were evaluated by a well-instructed researcher and an experienced neuro-radiologist in consensus. The tumor size was defined by the maximum tumor diameter in the transverse plane on both the initial and follow-up CE-T1WI and T2WI (Figure 1). As most imaging sequences were scanned in the axial plane and not in 3D mode, tumor growth was calculated in millimeters by subtracting the initial tumor diameter from the final tumor diameter measured on the first and the follow-up CE-T1WI and T2WI images. In order to correct for follow-up time, tumor growth was recalculated to growth percentage per year.

### 2.4. Statistical Analysis

A paired t-test was used to compare age, initial tumor size, absolute tumor growth, and average tumor growth (mm and percentage per year) between T2WI and CE-T1WI. The Wilcoxon signed-rank test was utilized to compare proportional growth rates between the imaging methods. A significance level of 0.05 was used to detect statistically significant differences. Sensitivity, specificity, positive predictive value (PPV), and negative predictive value (NPV) of T2WI were calculated. Statistical analysis was performed using IBM Statistical Package for the Social Sciences (IBM SPSS Statistics for Windows, Version 26.0. IBM Corp., Armonk, NY, USA).

## 3. Results

Ninety-nine eligible patients could be identified for retrospective data analysis based on the in- and exclusion criteria. The mean age of included patients was 59.9 years (range: 26–89 years). The mean radiological follow-up period was 1.9 years (range: 0.9–8.6 years), and the mean clinical follow-up period was 6.0 years (range: 0.9–11.7 years). Most meningiomas were located at the convexity (63%), followed by the falx (23%), infratentorial (9%) and skull base (5%). Thirteen patients underwent surgical resection of the monitored lesion, and all were revealed as meningiomas upon histopathological analysis. The average initial tumor diameter was 20.1 mm (SD = 11.8; range 5.2–63.5) on T2WI and 20.8 mm (SD = 12.1; range 6.3–72.1) on CE-T1WI (*p* < 0.001) (Table 1).

Tumor size was measured in the transverse plain on both T2WI and CE-T1WI of the MR scans upon initial presentation and during follow-up. Assuming tumors do not decrease in size over time and any measured decreases are due to imaging artifact, no tumor growth was found in 34.3% (*n* = 34) of patients on CE-T1WI and 37.4% (*n* = 37) of patients on T2WI. Thus, any increase in tumor size was found in 65.7% (*n* = 65) of the patients on T2WI and 62.6% (*n* = 62) of the patients on CE-T1WI, among which 16 patients had tumor growth ≥10% on T2WI and 10 on CE-T1WI (Table 1).

In patients with an increase in tumor size, the average tumor growth per year was 1.50 mm (SD= 2.2; range 0.06–15.7) for T2WI (*n* = 65) and 1.06 mm (SD = 1.08; range 0.02–6.13) for CE-T1WI (*n* = 62). The corresponding average percentage tumor growth per year was 8.6% (SD = 13.4; range 0.2–102) on T2WI and 9.5% (SD = 9.4; range 0.5–46) on CE-T1WI. Among those with clinically significant tumor growth of ≥10% per year, the average percentage growth of 22.6% (SD = 21; 10.6–102) was found on T2WI and 25.7% (SD = 11.8; 10.4–46) on CE-T1WI.

In the detection of (any) tumor growth, T2WI had a sensitivity of 79%, a specificity of 57%, a PPV of 75%, and an NPV of 62%, compared with the golden standard of CE-T1WI. In the detection of clinically significant tumor growth (≥10% per year), T2WI had a sensitivity of 80%, a specificity of 90%, a PPV of 47%, and an NPV of 98% compared to CE-T1WI. (Table 2).

## 4. Discussion

The current retrospective cohort study has demonstrated in a large sample size (*n* = 99) that a non-contrast T2WI was not inferior compared to CE-T1WI in the detection of radiological progression of asymptomatic meningiomas. Indeed, the detection of clinically significant meningioma growth (≥10% per year) was not missed in this cohort using T2WI. We found that T2WI overestimated the number of patients with tumor growth (*n* = 65 for T2WI vs. n = 62 for CE-T1WI). This may be explained by the statistically significant smaller tumor size upon initial T2WI, combined with thicker slices and different tilt at subsequent follow-up scans (the so-called partial volume effect). Taken together, these factors can result in small changes in the dimensions resulting in a larger tumor growth size.

The current study findings are in accordance with other retrospective studies that reported similar diagnostic accuracy of non-contrast T2WI with CE-T1WI in the long-term surveillance of asymptomatic meningiomas [9,10,11,12]. He et al. [9] also found T2WI images to overestimate growth. They also concluded that T2WI images were not inferior for follow-up imaging in retrospective patient cohorts of 18, 28, 82, and 122 patients [9,10,11,12]. In addition, T2WI has increasingly been found to be a reliable alternative to CE-T1WI in the surveillance of other CNS tumors, including glioma and vestibular schwannoma [13,14,15,16]. In the long-term surveillance of vestibular schwannoma, T2WI has been found to have equivalent diagnostic accuracy as CE-T1WI in growth detection and close to equivalent reliability in the visualization of intra-tumoral changes [17]. The use of thinner slices and sharper delineation from surrounding cerebrospinal fluid facilitates the identification of borders and therefore could lead to improvement of measurements.

Additionally, by limiting GBCA exposure, one could reduce Gd^3+^ deposition in the brain and minimize environmental pollution [18]. As Gd^3+^ is a rare metal and supply and availability is limited, reducing the need for GBCA for the radiological follow-up of meningiomas is not only sustainable for the environment but reduces costs and economic burden as well. Free gadolinium can be toxic and thus has to be chelated to ligands, which forms various GBCAs, broadly categorized as linear or macrocyclic [19]. Macrocyclic GBCAs are generally more stable due to the cage-like structure in which Gd^3+^ is enclosed [19] and are most frequently used nowadays. Chelated GBCAs should be cleared renally prior to destabilization, as the latter will result in the in vivo release of toxic Gd^3+^, resulting in deposits in the tissue of eyes, subcutaneous tissues, and skin, and organs such as the kidneys and brain [8,20,21]. Gd^3+^ deposition was found in the globus pallidus and dentate nucleus of otherwise healthy patients after undergoing repeated CE-T1WI imaging [11,20]. The clinical impact of gadolinium accumulation is scarcely investigated, and possible symptoms such as fatigue, imbalance, cognitive impairment, and headache have been described. However, expert opinion remains that the use of Gd^3+^ is non-toxic [21,22,23].

In clinical practice, tumor growth can generally be measured in multiple manners, such as 2D or 3D volume estimation. In this study, tumor diameters were measured in the transverse plane. A limitation of this technique is the 2D nature of objectifying tumor growth, as it does not take the possibility of meningioma growth in a different plane into account. Previous studies found suboptimal consensus between 2D diameter and 3D volumetric estimation of tumor dimensions and measurements of percentage growths in pediatric CNS tumors [24,25]. It has therefore been suggested that automated, semi-automated, or manual tumor volume measurement strategies may be more promising tools compared with manual 2D tumor diameter measurements for tumors with complex morphology [26,27,28]. However, not all radiology departments have the post-processing platforms and time needed for these 3D volumetrics, and this study aimed to evaluate the use of T2WI compared to CE-T1WI for evaluating meningioma growth in a clinically applicable and relevant setting. Three-dimensional volumes in multiplanar reformation could be used instead to assess tumor size in multiple planes.

Another limitation could be the duration of the mean follow-up time of this study (1.9 years). Meningioma-mimicking lesions such as lymphomas or dural metastases would have been distinguished during the clinical follow-up period of this study (mean is 6.0 years). The main objective of this study was to determine if T2WI was non-inferior to CE-T1WI for the detection of significant tumor growth (≥10% per year). Furthermore, meningioma growth varies in an exponential, linear, and deaccelerating pattern during a long follow-up time [29,30,31,32]. Notably, more than 40% of meningiomas show the most growth over a period of four years after the initial diagnosis [4]. Indeed, this study showed that, in a large dataset, T2WI is at least as accurate as CE-T1WI in the detection of significant meningioma growth.

## 5. Conclusions

The use of T2WI could be used as the standard imaging modality for follow-up of meningioma in asymptomatic patients, albeit followed by post-contrast imaging in case of doubt or growth. Furthermore, additional studies could focus on non-contrast 3D sequences to optimize assessment of radiological tumor growth and volume.

## Figures and Tables

**Table 1:** Average tumor diameter and average tumor growth in millimeters and percentage on T2WI and CE-T1WI (*n* = 99).

Parameters	T2WI	CE-T1WI	*p* Value
Initial tumor diameter (mm (SD; range))	20.1 (11.8; 5.2–63.5)	20.8 (12.1; 6.3–72.1)	<0.001
Tumor growth (mm (mean (SD; range))	0.99 (2.96; −7.1–17.2)	0.53 (2.48; −11.6–10.4)	0.057
Average tumor growth in millimeters (mm/year (SD; range))	0.69 (2.2; −3.7–15.7)	0.26 (1.8; −10.6–6.1)	0.076
Average tumor growth in percentage (%/year (SD; range))	4.1 (12.7; −13.3–102.4)	1.8 (7.1; −17.9–21.7	0.038
Proportional tumor growth (*n* (%))			
None	34 (34.3)	37 (37.4)	0.460
<10%	49 (49.5)	52 (52.5)	
≥10%	16 (16.2)	10 (10.1)	

**Table 2:** Sensitivity, specificity, PPV, and NPV of MRI T2WI in detection of any tumor growth compared to CE-T1WI (middle column) and ≥10% yearly tumor growth (right column).

	Any Yearly Tumor Growth	Yearly Tumor Growth ≥ 10%
Sensitivity	79.0%	80.0%
Specificity	56.8%	89.9%
PPV	75.4%	47.1%
NPV	61.8%	97.6%

## Data Availability

Data is available upon reasonable request.

## Abbreviations

The following abbreviations are used in this manuscript:

CE-T1WI	Contrast-Enhanced T1-Weighted Images
CNS	Central nervous system
GBCA	Gadolinium-Based Contrast Agents
Gd^3+^	Gadolinium
NPV	Negative Predictive Value
PPV	Positive Predictive Value
SD	Standard deviation
T1WI	T1-Weighted Images
T2WI	T2-Weighted Images
